# Serum sCD25 Protein as a Predictor of Lack of Long-Term Benefits from Immunotherapy in Non-Small Cell Lung Cancer: A Pilot Study

**DOI:** 10.3390/cancers13153702

**Published:** 2021-07-23

**Authors:** Anna Siemiątkowska, Maciej Bryl, Katarzyna Kosicka-Noworzyń, Jakub Tvrdoň, Iwona Gołda-Gocka, Aleksander Barinow-Wojewódzki, Franciszek K. Główka

**Affiliations:** 1 Department of Physical Pharmacy and Pharmacokinetics, Poznan University of Medical Sciences, 6 Święcickiego Street, 60-781 Poznań, Poland; kasiakosicka@ump.edu.pl (K.K.-N.); jakub.tvrdon.pl@gmail.com (J.T.); glowka@ump.edu.pl (F.K.G.); 2 Department of Pharmaceutics, Ernest Mario School of Pharmacy, Rutgers, The State University of New Jersey, 160 Frelinghuysen Road, Piscataway, NJ 08854, USA; 3 Center of Excellence for Pharmaceutical Translational Research and Education, Ernest Mario School of Pharmacy, Rutgers, The State University of New Jersey, 160 Frelinghuysen Road, Piscataway, NJ 08854, USA; 4 Department of Clinical Oncology with the Subdepartment of Diurnal Chemotherapy, Wielkopolska Center of Pulmonology and Thoracic Surgery, 62 Szamarzewskiego Street, 60-569 Poznań, Poland; mbryl@wcpit.org (M.B.); agocki@wp.pl (I.G.-G.); awojewodzki@wcpit.org (A.B.-W.)

**Keywords:** biomarkers, sCD25, atezolizumab, pembrolizumab, anti-PD-1/PD-L1

## Abstract

**Simple Summary:**

The prognosis of advanced lung cancer is poor. Even though it can improve with immunotherapy, most patients do not respond to treatment. Identifying patients who would not benefit from therapy is an unmet goal. We hypothesized that one of the molecules present in human serum (namely, the soluble form of the unit α of the interleukin-2 receptor, sCD25) could be used as a predictor of successful immunotherapy in patients with lung cancer. Our study showed that patients who presented high sCD25 levels before treatment (≥5.99 ng/mL) and/or about three months from the start of treatment (≥7.73 ng/mL) progressed faster and lived shorter without the disease progression and serious toxicity. Serum levels of sCD25 could easily indicate patients with lung cancer who would not achieve long-term benefits from immunotherapy. Therefore, other more effective therapies could be implemented.

**Abstract:**

Prognosis of advanced non-small cell lung carcinoma (NSCLC) is poor. Even though it can improve with anti-PD-1/PD-L1 agents, most patients do not respond to treatment. We hypothesized that the serum soluble form of the unit α of the interleukin-2 receptor (sCD25) could be used as a biomarker of successful immunotherapy in NSCLC. We recruited patients dosed with atezolizumab (*n* = 42) or pembrolizumab (*n* = 20) and collected samples at baseline and during the treatment. Levels of sCD25 were quantified with the ELISA kits. Patients with a high sCD25 at baseline (sCD25.0 ≥ 5.99 ng/mL) or/and at the end of the fourth treatment cycle (sCD25.4 ≥ 7.73 ng/mL) progressed faster and lived shorter without the disease progression and serious toxicity. None of the patients with high sCD25 at both time points continued therapy longer than 9.3 months, while almost 40% of patients with low sCD25 were treated for ≥12.3 months. There was a 6.3-times higher incidence of treatment failure (HR = 6.33, 95% CI: 2.10–19.06, *p* = 0.001) and a 6.5-times higher incidence of progression (HR = 6.50, 95% CI: 2.04–20.73, *p* = 0.002) in patients with high compared with low sCD25.0 and sCD25.4. Serum levels of sCD25 may serve as a non-invasive biomarker of long-term benefits from the anti-PD-1/PD-L1s in NSCLC.

## 1. Introduction

Non-small-cell lung carcinoma (NSCLC) comprises 80–85% of all lung cancers. Usually, there are no symptoms until the disease is at an advanced stage. Thus, diagnosis is often late [1]. The five-year survival of lung cancer patients in Europe between 1999 and 2007 was about 13% [2], while in the U.S., it was about 17% in 2009 [3]. The introduction of immune-checkpoint inhibitors (ICIs) reduced the mortality rate by approx. 30% [4]. However, the response rates are still below expectations. Depending on the subset of NSCLC patients, the highest observed overall response rates (ORRs) range between 30 and 40% [5,6,7,8]. Importantly, some patients benefit from treatment only temporarily [9,10]. Thus, non-responders, short-term responders, and long-term survivors can be distinguished among patients dosed with the ICIs [11].

The programmed cell death protein 1 (PD-1) receptor, or its ligand 1 (PD-L1) directed agents became crucial in the treatment of NSCLC including atezolizumab (ATEZO), nivolumab (NIVO), and pembrolizumab (PEMBRO) [4,8]. They inhibit the interaction between PD-1 (located on T cells) and PD-L1 (expressed by tumor cells). Such an action prevents the immune response inhibition and blocks the mechanism used by tumor cells to evade the immune eradication [12]. A correlation between PD-L1 tumor expression and overall survival (OS) or progression-free survival (PFS) has been established [6] and, up to now, PD-L1 expression is the only biomarker used in clinical practice for patients’ qualification for ICI therapy. However, several clinical trials have shown that patients with no PD-L1 tumor expression also benefited from immunotherapy [7,13]. Based on the PD-L1 expression, it is impossible to indicate which patients would not benefit from therapy [5]. The predictive power of PD-L1 is limited by the heterogeneity of its expression within the tumor tissue and the diversity of antibodies used in the assays [5,14,15].

Great emphasis is being placed on understanding why only a subset of patients responds to immunotherapy. Efforts are being made to develop a panel of biomarkers that could identify patients that would most likely benefit from ICIs [16]. Recent meta-analyses have shown that particularly useful in this field could be tumor mutation burden [17] and neutrophil-to-lymphocyte ratio [18] that impacted both OS and PFS in NSCLC patients treated with ICIs.

Several soluble proteins have been identified to correlate with immune activation, (e.g., a soluble form of the unit α of interleukin-2 (IL-2) receptor, called sCD25 or sIL-2Rα) [19]. The sCD25 was postulated as a surrogate marker of T-cell activation and the indicator of subsequent cellular death [20]. The exact role of sCD25 in immunity and tolerance mechanisms remains unclear, as summarized recently by Damoiseaux [21]. One of the hypotheses concerning the function of sCD25 says that it could act as a decoy receptor for IL-2, resulting in reduced bioavailability of IL-2. An intermediate-affinity dimeric receptor (units β and γ) expressed by NK-cells and conventional T-cells would then lose the competition for IL-2 with a high-affinity tri-molecular receptor (units α, β, and γ) expressed by regulatory T cells (Tregs). As a consequence, tolerance induction would be favored over the activation of T-cell responses [21].

The elevated levels of serum sCD25 (sIL-2R) have been linked with poor outcomes in various blood [22,23] and solid cancers [24,25,26,27], including NSCLC [28,29,30]. Cabrera and colleagues [25,31] observed the elevated levels of sCD25 in hepatocellular carcinoma and postulated sCD25 as a mediator of T-cell suppression and tumor progression. Due to their mechanism of action, ICIs-based drug regimens also started to be investigated for their efficacy in relation to the sCD25 levels. An in vitro study showed that sCD25 protein inhibited the antitumor effects of cytotoxic T lymphocyte antigen-4 (CTLA-4) blockade [32]. Accordingly, higher baseline sCD25 predicted shorter OS in metastatic melanoma patients administered with different anti-CTLA-4 agents [32,33].

Currently, there are no data regarding sCD25 levels in NSCLC patients treated with ICIs. This study aimed to assess the possible utility of sCD25 in lung cancer as an early indicator of the long-lasting benefits and durable responses to the anti-PD-1/PD-L1 agents. We assessed the protein levels in sera of patients with advanced NSCLC, both at the initiation of the therapy with ATEZO or PEMBRO and during subsequent doses.

## 2. Materials and Methods

### 2.1. Study Population

This study included 62 individuals with advanced NSCLC, qualified to the national drug program (Supplementary Table S1) and treated with ATEZO (*n* = 42) or PEMBRO (*n* = 20) in the Eugenia and Janusz Zeyland Wielkopolska Center of Pulmonology and Thoracic Surgery in Poland. Patients received the drug in monotherapy as a first-line (PEMBRO; 200 mg Q3W) or a second-line treatment (ATEZO; 1200 mg Q3W). We recruited patients before their first anti-PD-1/PD-L1 administration and asked them to provide five blood samples, the first one at enrollment (baseline; sample taken just before the first dose of ICI), and the next samples in three-week intervals, just before administrating the consecutive doses of ICI (the last sample was taken at the end of cycle four, just before administrating the fifth dose). Immunotherapy was continued until unacceptable toxicity, disease progression, or death. Each patient filled out a questionnaire on anthropometric measurements, general health, and smoking habits.

Response to treatment was assessed every three months according to the Response Evaluation Criteria in Solid Tumors (RECIST, version 1.1) [34], and was classified as complete response (CR), partial response (PR), stable disease (SD), or progressive disease (PD). Patients with PD were no longer eligible for immunotherapy and stopped receiving the anti-PD-1/PD-L1s. Each patient who continued therapy was followed up for at least twelve months from the start of treatment. Clinical benefits (disease control) were defined as obtaining SD, PR, or CR and objective response (OR) as obtaining PR or CR. PFS was measured as the time from the initiation of therapy to confirmed progression (PD or death); time to treatment failure (TTF) as the time from the initiation of therapy to its discontinuation; and OS as the time from the start of therapy to death.

BMI and smoking status were calculated based on the self-reported data; never smokers were defined as patients who smoked <100 cigarettes in their lifetime, and former/current smokers as patients who smoked ≥100 cigarettes and had quit smoking (former smokers) or smoked cigarettes (current smokers) at the time of the interview [35].

Table 1 presents the detailed characteristics of the study population. Thirteen out of sixty-two patients (21.0%) were still on treatment at the time of data cut-off (TTF ≥ 12.9 months). After a minimum follow-up of twelve months, the ORR was 16.1%. Half of the patients discontinued therapy within 5.7 months from the start of treatment (Supplementary Figure S1A), and the main reason for the end of therapy was confirmed progression (Supplementary Figure S1B). In total, 11 patients were not evaluated for any treatment response, including those who died before the first response evaluation. During the first three months of immunotherapy (i.e., at the time of sample collection), the next dose of ICI was postponed at least one time in 28 patients (45.2%), and the median delay time was four days. The delay was usually due to the patient’s condition, but sometimes due to medical-unrelated issues. For a few patients, the blood sample collection was disrupted as a result of the COVID-19 pandemic and hospital restrictions. Therefore, the number of samples coming from these patients was incomplete. In total, 249 blood samples were collected and used in the analyses.

### 2.2. Samples and Methods

The peripheral blood samples were centrifuged at 1700× *g* for 15 min, aliquoted, and stored at −80 °C until use. Serum sCD25 was determined by the sandwich enzyme-linked immunosorbent assay (ThermoFisher Scientific, Waltham, MA, USA; Cat. No. BMS212-2), strictly according to the manufacturer’s protocol. The optical density was read at 450 nm with reference 630 nm with the BioTek 800TS plate reader (BioTek Instruments, Winooski, VT, USA). Each sample was analyzed in duplicate. A log-log linear regression was used to calculate the sCD25 levels.

### 2.3. Statistics

All statistical analyses were performed with Statistica 13.3 software (Statsoft Inc., Tulsa, OK, USA). The Shapiro–Wilk test was used to check for normal distribution of continuous variables. Normally distributed data were then expressed as mean ± sd, while skewed data as median (interquartile range, IQR). Qualitative data were expressed as numbers and corresponding percentages. For between-group comparisons, the *t*-Student test, Mann–Whitney U test, Kruskal–Wallis test, or Chi-square test were used, as appropriate. The changes in biomarker levels over time compared to baseline were assessed with the Wilcoxon test. The Kaplan–Meier curves were used to check the associations between high and low protein levels and OS, PFS, and TTF. The differences between the curves were calculated with the log-rank test, and patients who did not experience the event of interest (death for OS analyses; progression or death for PFS analyses; treatment failure for TTF analyses) were censored in Kaplan–Meier analyses. Patients who stopped immunotherapy due to other reasons than progression or death were censored in PFS analyses unless they died within the three months after discontinuation of immunotherapy. Such an approach was necessary as these patients were lost to follow-up. The Cox proportional hazard models were used to confirm the prognostic role of the tested biomarkers on OS, PFS, and TTF. For all performed analyses, a *p*-value < 0.05 was considered significant.

## 3. Results

### 3.1. Kinetic Changes in Serum sCD25

**Figure 1:**
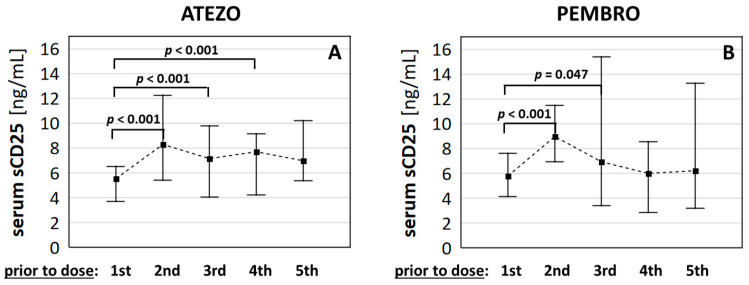
Changes in serum sC25 during the first four cycles of immunotherapy with anti-PD-1/PD-L1 drugs. Figure presents medians (filled squares) and interquartile ranges (whiskers) of sCD25 protein in subjects with NSCLC receiving (**A**) ATEZO and (**B**) PEMBRO.

We started our analyses by testing how sCD25 levels changed after the start of immunotherapy. The sCD25 level increased after the first dose of ATEZO in all patients, and PEMBRO in 88.2% of patients. The relative change compared to baseline (sCD25.1/0 ratio) was 154.5% (136.6–205.3) in ATEZO and 147.7% (119.5–208.2) in PEMBRO subgroup (medians (IQRs)). It was the highest increase in the sCD25 level during the first three months of immunotherapy in both groups (Figure 1A,B). We did not find any significant differences in sCD25 levels between patients dosed with ATEZO and PEMBRO at any of the evaluated time points. Therefore, further analyses were performed after combining these patients into one study group.

### 3.2. Association with Clinicodemographic Data

The sCD25 levels and their changes were independent of the NSCLC subtype (Supplementary Figure S2), smoking status (Supplementary Figure S3), or BMI< and ≥ 25kg/m^2^ (Supplementary Figure S4). Serum sCD25 did not differ significantly between females and males (Supplementary Figure S5A1–A5). However, the relative changes of sCD25 across therapy tended to be higher in females than males, and the difference reached significance for sCD25.1/0, sCD25.2/0, and sCD25.4/0 (Supplementary Figure S5B1–B4). Older patients (≥ 65 years old) presented a stronger increase in sCD25 levels at the end of cycle 1 (sCD25.1/0 ratio) (Supplementary Figure S6).

Due to revealed differences, further analyses were performed with sCD25 levels only (sCD25.0–sCD25.4), not with their relative changes compared to baseline (sCD25.1/0–sCD25.4/0).

### 3.3. Clinical Benefits 1-Year from the Start of Immunotherapy

**Figure 2:**
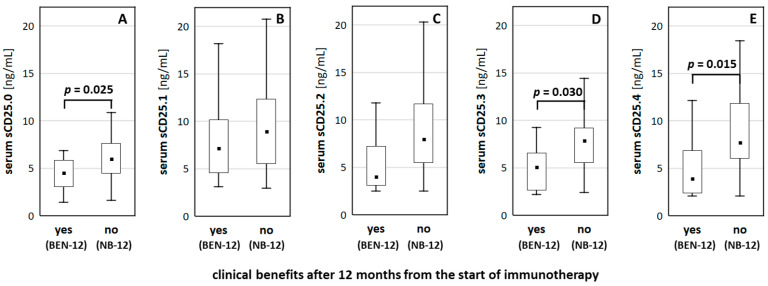
Serum sCD25 levels stratified by achieving long-term benefits from treatment. Figure presents medians (filled squares), interquartile ranges (boxes), and ranges excluding outliers for sCD25 protein measured at (**A**) baseline and at the end of (**B**) cycle 1, (**C**) cycle 2, (**D**) cycle 3, and (**E**) cycle 4 in patients who obtained (BEN-12) and lacked (NB-12) clinical benefits after 12 months from the start of immunotherapy.

Baseline serum sCD25 concentrations (sCD25.0) were significantly lower in patients who presented disease control after twelve months from the start of immunotherapy (BEN-12 group) than in those with no benefits (NB-12 group): 4.50 (3.03–5.82) vs. 5.99 (4.45–7.64) ng/mL (Figure 2A). After the first dose of the anti-PD-1/PD-L1s, the sCD25 level increased significantly in both groups (*p* < 0.001 from Wilcoxon test). However, it returned to baseline values only in BEN-12 group (*p* > 0.05 for sCD25.3 and sCD25.4 levels compared to baseline). The levels of sCD25 were significantly lower in BEN-12 than in NB-12 group at the end of cycle three (sCD25.3) and four (sCD25.4): 5.07 (2.67–6.58) vs. 7.89 (5.53–9.20) ng/mL for sCD25.3 (Figure 2D) and 3.92 (2.38–6.85) vs. 7.73 (6.05–11.83) ng/mL for sCD25.4 (Figure 2E).

### 3.4. Survival Analyses

Based on the previous analysis, levels of sCD25.0, sCD25.3, and sCD25.4 were chosen for survival analyses. The cut-offs were median values for patients without long-term benefits (assessed after twelve months from the initiation of therapy; NB-12 group). For biomarkers that differed significantly in the survival curves, Cox regression analysis was performed to account for other factors’ influence.

The log-rank test indicated that sCD25 level was not predictive in terms of patient survival (OS). Patients stratified according to the sCD25.0 (cut-off: 5.99 ng/mL) and sCD25.4 concentrations (cut-off: 7.73 ng/mL) differed in TTF (*p* = 0.008 and *p* = 0.013) and PFS (*p* = 0.019 and *p* = 0.023, respectively).

**Figure 3:**
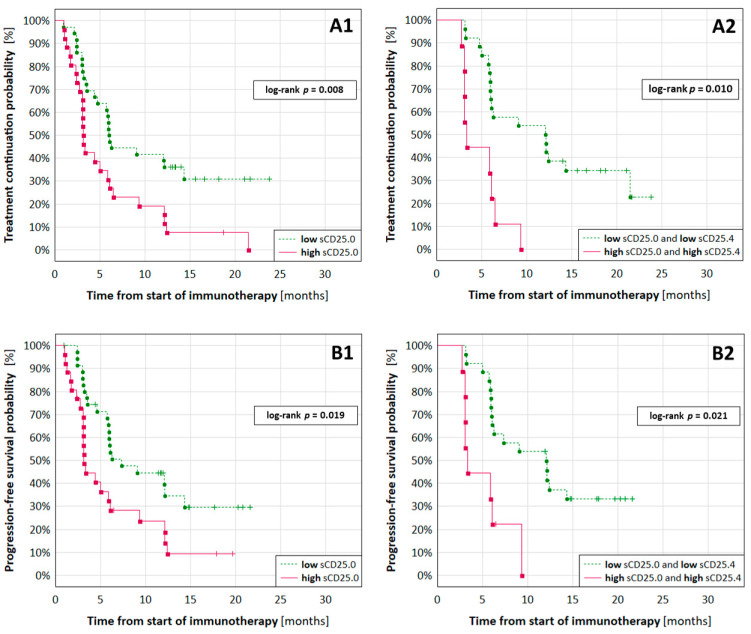
Kaplan–Meier curves for TTF and PFS based on the sCD25 levels. High sCD25.0 level (≥5.99 ng/mL) was a prognostic factor of (**A1**) shorter TTF and (**B1**) shorter PFS; high sCD25.0 (≥5.99 ng/mL) and sCD25.4 (≥7.73 ng/mL) levels were a prognostic factor of (**A2**) shorter TTF and (**B2**) shorter PFS in NSCLC patients dosed with ATEZO or PEMBRO; complete observations are denoted as filled circles, censored observations as crosses.

The median values for TTF in patients with low and high sCD25.0 were 6.0 and 3.1 months (Figure 3A1), and for PFS it was 6.3 and 3.1 months (Figure 3B1). A lack of short-term benefits from treatment was observed in 27.8% of patients with low and 57.7% of patients with high baseline sCD25 (Table 2). After a year, the therapy was continued by 36.1% of patients with low baseline and 7.7% of patients with high baseline sCD25 (Table 2).

The median values for TTF in patients with low and high sCD25.4 were 12.1 vs. 4.7 months, and for PFS it was 12.1 vs. 5.9 months. Three months from the start of therapy, a lack of clinical benefits from treatment was observed in 4.8% of patients with low and 42.9% of patients with high sCD25.4. The therapy was continued for at least a year by 42.9% of patients with low and 7.1% of patients with high sCD25.4 (Table 2).

Figure 3A2,B2 present the differences in TTF and PFS after stratifying patients by high and low levels of both sCD25.0 and sCD25.4. None of the patients with high sCD25.0 and sCD25.4 continued the therapy longer than 9.3 months; almost 40% of patients with low sCD25.0 and sCD25.4 were treated for at least 12.3 months (Figure 3A2).

The univariate Cox regression showed that PFS and TTF were independent on gender, administered drug, smoking status, age, BMI, and NSCLC subtype. High sCD25.0 (≥5.99 ng/mL) was a prognostic factor of shorter TTF (HR = 2.18, 95% CI: 1.24–3.83, *p* = 0.007) and PFS (HR = 2.06, 95% CI: 1.14–3.74, *p* = 0.017); high sCD25.4 (≥7.73 ng/mL) was a prognostic factor of shorter TTF (HR = 2.93, 95% CI: 1.34–6.40, *p* = 0.007) and PFS (HR = 2.76, 95% CI: 1.22–6.23, *p* = 0.014); high concentrations of both sCD25.0 (≥5.99 ng/mL) and sCD25.4 (≥7.73 ng/mL) were a prognostic factor of shorter TTF (HR = 4.18, 95% CI: 1.73–10.08, *p* = 0.001) and PFS (HR = 3.90, 95% CI: 1.56–9.76, *p* = 0.004).

After stratifying patients by low and high levels of sCD25.0, sCD25.4 or both, between-group comparison with regard to patients’ characteristics was additionally performed to confirm groups’ similarity. Most patients with a low concentration of sCD25.0 were ≥65 years old (72.2% vs. 27.8%, *p* < 0.05), and a similar trend was observed for a group with low both sCD25.0 and sCD25.4 (69.2% vs. 30.8%, *p* = 0.068). Moreover, in a high sCD25.0 level subgroup, there was a trend toward more males than females (73.1 vs. 26.9%, *p* = 0.058). No other between-group differences were observed (data not shown).

To account for these discrepancies, the multivariable Cox regression analysis was performed that included (1) age ≥65 and gender and as the confounding factors in sCD25.0 analyses and (2) age ≥65 in the analyses including both sCD25.0 and sCD25.4. High sCD25.0 (≥5.99 ng/mL) was an independent prognostic factor of shorter TTF (HR = 2.34, 95% CI: 1.22–4.48, *p* = 0.010) and PFS (HR = 2.24, 95% CI: 1.13–4.44, *p* = 0.021); high concentrations of both sCD25.0 (≥5.99 ng/mL) and sCD25.4 (≥7.73 ng/mL) were an independent prognostic factor of shorter TTF (HR = 6.33, 95% CI: 2.10–19.06, *p* = 0.001) and PFS (HR = 6.50, 95% CI: 2.04–20.73, *p* = 0.002).

## 4. Discussion

ICIs have improved the treatment outcomes in various cancers, including NSCLC [4]. However, not all patients benefit from the therapy [6,36], which raises economic and ethical concerns. Here, we demonstrate that a high level of serum sCD25 could be a prognostic factor of lack of short and long-term benefits from treatment with ATEZO or PEMBRO. Patients with a high sCD25 level at baseline (sCD25.0) or/and at the end of the fourth cycle of drug dosing (sCD25.4) progressed faster and lived shorter without the disease progression and/or serious toxicity.

The sCD25 molecule has been studied for many years, mainly in conditions associated with the dysregulated immune system. Serum sCD25 is elevated in various autoimmune and inflammatory conditions but also in neoplastic diseases [21], including lung cancer. Several authors have reported higher levels of sCD25 in NSCLC [29,37,38,39,40,41], and our results are in line with these observations. Baseline sCD25 (sCD25.0) in our population was 5.62 (3.72–6.88) ng/mL (median, IQR), while the literature data indicate the mean or median values for healthy volunteers was in the range of 0.45–3.83 ng/mL [39,42,43,44].

We did not find any significant differences in sCD25 levels between patients with ADC and SQC, which supports the observations presented by others [37,38,39,40]. The sCD25 levels were also independent on gender, though the relative changes in sCD25 compared to baseline were or tended to be higher in females than in males. Data from the literature show that while the sCD25 protein level is not affected by gender in healthy individuals [45,46], it may be disease-specific [42]. Although some authors reported that serum sCD25 increased with age [27,46,47], others obtained contradictory results [22,42]. We observed no difference in baseline sCD25 between older and younger individuals. However, after the first dose of ICI, sCD25 level increased more in older (≥65 years) than in younger patients (163.2 vs. 137.8%, *p* < 0.01). In general, serum sCD25 increased at the end of the first treatment cycle almost in all patients dosed with ATEZO or PEMBRO, and this agrees with the observations for other ICIs [32,48].

Reports about sCD25 and ICIs concern mostly CTLA-4-blockade and melanoma patients [32,33]. A study by Hannani and colleagues [32] revealed that higher baseline sCD25 predicted shorter OS in patients administered with ipilimumab. Similar conclusions were later presented by Bajor and colleagues [33] for concomitant therapy with tremelimumab and a CD40 agonist. We did not confirm the association between sCD25 values and OS in the study population. However, we demonstrated that the concentrations of sCD25 measured at selected time points during immunotherapy were significant predictors of TTF and PFS. One year after the start of the anti-PD-1/PD-L1 treatment, at least three times more NSCLC patients did not experience progression, and about four times more patients still continued therapy when their baseline sCD25 was low (<5.99 ng/mL). This corroborates with the results of Armand and colleagues [49], who reported poor response to NIVO (anti-PD-1) in patients with lymphoma and high baseline sCD25.

We also noticed that levels of sCD25 measured at the end of the fourth cycle of treatment (i.e., about three months from the start of immunotherapy) were predictive in terms of achieving clinical benefits. The relationship was even stronger when both time points were considered. All patients with high levels of sCD25.0 and sCD25.4 lacked long-term benefits, while almost 40% of patients with low levels achieved long-term benefits from treatment. Importantly, other authors highlighted a better response to the anti-PD-1 agents among NSCLC patients presenting higher IL-2 levels three months from the start of treatment [50]. Together, these observations support the hypothesis on the role of sCD25 as a decoy receptor for IL-2, favoring Tregs and tolerance induction and depriving effector T-cells of sufficient IL-2 [21].

Recently, Takai and colleagues [51] linked high levels of sCD25 with various immune-related adverse events in patients with malignant diseases dosed with ICIs. Moreover, Yoshida and colleagues [52] showed that sCD25 predicted the occurrence and the disease activity of the checkpoint inhibitor pneumonitis in NSCLC. In our population, only a small number of patients experienced severe (at least grade 3) toxicity (Supplementary Figure S1B). Thus, we did not correlate serum sCD25 with the immune-related adverse events. However, we noticed that in survival analyses, high sCD25 levels usually impacted to a higher extent TTF than PFS, suggesting that not all patients with higher sCD25 levels discontinued therapy due to progression.

In the literature, there is no agreement on whether the sCD25 levels reflect disease progression in NSCLC. Most, but not all [29,53], studies have shown no differences in the concentrations of sCD25 between different stages of NSCLC [38,39,41]. We demonstrated that high baseline sCD25 predicted not only short (three months) but also long-term benefits from immunotherapy (twelve months). Therefore, worse outcomes in the high sCD25 level groups resulted more likely from the poor anti-PD-1/PD-L1 efficacy rather than disease progression. To our best knowledge, our study is the first one indicating sCD25 as a useful marker that could be easily measured and indicate patients with NSCLC who would not benefit from immunotherapy with ATEZO or PEMBRO. Worth noting is that we obtained a similar cut-off for baseline sCD25 (5.99 ng/mL) as Cabrera et al. [25] (5.80 ng/mL) and Wang et al. [22] (6.05 ng/mL), who found correlations between the elevated sCD25 levels and shorter OS or PFS in other types of cancers.

The source of the elevated sCD25 levels in NSCLC patients undergoing immunotherapy remains unclear. However, it is probably not derived from the tumor microenvironment. In patients with rare solid tumors, exposure to the anti-PD-1 agent resulted in decreased secretion of sIL-2R in CD8+ tumor-infiltrating lymphocytes [54]. The source of the sCD25 could be the tumor tissue. Indeed, Yano et al. [53] showed that lung cancer cells express CD25 antigen and may release its soluble form. Moreover, sCD25 might also be released from the activated peripheral T cells, e.g., Tregs [55]. In line with this hypothesis, in NSCLC, non-responders to PD-1 blockade presented a higher percentage of baseline CD25+FOXP3+CD4+ T cells [11].

The strengths of the project include its prospective character in terms of sample collection. We collected samples both prior to and after the start of immunotherapy, which allowed for assessing how the levels of sCD25 changed across the treatment. Moreover, all NSCLC patients were undergoing monotherapy with ATEZO or PEMBRO with the same dosing regimen. This allowed for better evaluation of how the anti-PD-1/PD-L1 drugs relate to the levels of sCD25 regardless of the other treatment options. The major limitation of our study is the small sample size. This project was a one-year pilot study conducted in one of the local hospitals. Therefore, the number of participants was relatively small. We aimed to assess whether the soluble sCD25 protein might have a predictive value in indicating patients who would benefit from immunotherapy, which could justify further studies. Additionally, the immunotherapy was temporarily discontinued in a few patients. Thus, the time between the next doses and sample collection sometimes exceeded 21 days. This interruption could affect the results but was impossible to avoid in real-world settings. Finally, though both drugs blocked the PD-1/PD-L1 pathway, PEMBRO was administered as a first-line treatment while ATEZO was administered as a second-line treatment. This discrepancy was due to eligibility criteria for the NSCLC drug program in Poland at the time of conducting the study (Supplementary Table S1).

## 5. Conclusions

To sum up, we demonstrated that high levels of serum sCD25 protein at baseline and three months from the start of immunotherapy could easily and quickly identify NSCLC patients who would not achieve long-term benefits from ATEZO or PEMBRO treatment. This observation supports the hypothesis on the benefits from monitoring anticancer therapy with serum sCD25 [21].

## Figures and Tables

**Table 1:** Characteristics of NSCLC patients treated with anti-PD-1/PD-L1 agents.

	All (*n* = 62)	ATEZO (*n* = 42)	PEMBRO (*n* = 20)	*p*-Value
• Age [years]	65.4 ± 7.2	65.1 ± 6.3	66.2 ± 8.9	NS
- age ≥ 65	37 (59.7%)	26 (61.9%)	11 (55.0%)	NS
• BMI [kg/m^2^]	25.8 ± 4.3	26.4 ± 4.3	24.5 ± 4.1	NS
- BMI ≥ 25	30 (48.4%)	21 (50.0%)	9 (45.0%)	NS
- missing information	3 (4.8%)	3 (7.1%)	0 (0.0%)	
• Gender				
- females	25 (40.3%)	15 (35.7%)	10 (50.0%)	NS
- males	37 (59.7%)	27 (64.3%)	10 (50.0%)	
• Smoking status				
- never smoker	7 (11.3%)	4 (9.5%)	3 (15.0%)	NS
- former smoker	39 (62.9%)	28 (66.7%)	11 (55.0%)	
- current smoker	13 (21.0%)	7 (16.7%)	6 (30.0%)	
- missing information	3 (4.8%)	3 (7.1%)	0 (0.0%)	
• Type of NSCLC				
- ADC	38 (61.3%)	27 (64.3%)	11 (55.0%)	NS
- SQC	19 (30.6%)	11 (26.2%)	8 (40.0%)	
- NOS/other/missing information	5 (8.1%)	4 (9.5%)	1 (5.0%)	
• Stage of NSCLC				
- III *	2 (3.2%)	2 (4.8%)	0	NS
- IV	60 (96.8%)	38 (95.2%)	20 (100%)	
• Best response to treatment ^#^				
- CR	0	0	0	NS
- PR	10 (16.1%)	4 (9.5%)	6 (30.0%)	
- SD	26 (41.9%)	17 (40.5%)	9 (45.0%)	
- PD	23 (37.1%)	18 (42.8%)	5 (25.0%)	
- *n*/e	3 (4.8%)	3 (7.1%)	0	

Continuous (normally distributed data) were expressed as mean ± sd, categorical data were expressed as *n* (%); * III B T4N2M0; ^#^ death before the first treatment evaluation was considered PD. Abbreviations: ADC, adenocarcinoma; ATEZO, atezolizumab; BMI, body mass index; CR, complete response; *n*/e, not evaluated; NOS, not otherwise specified; NS, not significant; NSCLC, non-small cell carcinoma; PD, progressive disease; PEMBRO, pembrolizumab; PR, partial response; SD, stable disease; SQC, squamous cell carcinoma.

**Table 2:** Clinical benefits in NSCLC patients treated with ATEZO or PEMBRO.

		Clinical Benefits 3 Months from the Start of Treatment			Clinical Benefits 12 Months from the Start of Treatment		
		Yes	No	*p*-Value	Yes	No	*p*-Value
sCD25.0	low	26 (72.2%)	10 (27.8%)	*p* < 0.05	13 (36.1%)	23 (63.9%)	*p* < 0.05
	high	11 (42.3%)	15 (57.7%)		2 (7.7%)	24 (92.3%)	
sCD25.4	low	20 (95.2%)	1 (4.8%)	*p* < 0.01	9 (42.9%)	12 (57.1%)	*p* < 0.05
	high	8 (57.1%)	6 (42.9%)		1 (7.1%)	13 (92.9%)	
sCD25.0 & sCD25.4	low	24 (92.3%)	2 (7.7%)	*p* < 0.01	10 (38.5%)	16 (61.5%)	*p* < 0.05
	high	4 (44.4%)	5 (55.6%)		0 (0%)	9 (100%)	

Results are presented as number of patients (%); sCD25.0 (cut-off: 5.99 ng/mL) and sCD25.4 (cut-off: 7.73 ng/mL) levels of sCD25 at baseline and at the end of cycle four.

## Data Availability

All data relevant to the article were included in the manuscript and presented as Tables and Figures, or were provided as Supplementary Materials. Additional data are available upon reasonable request from the corresponding author.

## Supplementary Materials

The following are available online at https://www.mdpi.com/article/10.3390/cancers13153702/s1: Figure S1, (A) Time to treatment failure and (B) reason for ending the anti-PD-1/PD-L1 treatment in patients with non-small cell lung carcinoma dosed with atezolizumab or pembrolizumab; Figure S2, Serum sCD25 levels and their relative changes stratified by NSCLC subtype; Figure S3, Serum sCD25 levels and their relative changes stratified by smoking status; Figure S4, Serum sCD25 levels and their relative changes stratified by BMI; Figure S5, Serum sCD25 levels and their relative changes stratified by gender; Figure S6, Serum sCD25 levels and their relative changes stratified by age; Table S1, Eligibility criteria for treatment with ATEZO or PEMBRO, according to the NSCLC drug program in Poland at the time of conducting the study.

## Abbreviations

ATEZO	Atezolizumab
BMI	body mass index
CR	complete response
CTLA-4	cytotoxic T lymphocyte antigen-4
ICI	immune-checkpoint inhibitor
IL-2	interleukin-2
IQR	interquartile range
NIVO	nivolumab
NSCLC	non-small-cell lung carcinoma
OR	objective response
ORR	objective response rate
OS	overall survival
PD	progressive disease
PD-1	programmed cell death protein 1
PD-L1	programmed cell death ligand 1
PEMBRO	pembrolizumab
PFS	progression-free survival
PR	partial response
RECIST	Response Evaluation Criteria in Solid Tumors
sCD25	soluble form of the unit α of interleukin-2 receptor (sIL-2Rα)
sd	standard deviation
SD	stable disease
Tregs	regulatory T cells
TTF	time to treatment failure
