# Supplementary material for: Serum sCD25 Protein as a Predictor of Lack of Long-Term Benefits from Immunotherapy in Non-Small Cell Lung Cancer: A Pilot Study
Source: Cancers (Basel). 2021 Jul 23;13(15):3702. doi: 10.3390/cancers13153702 (PMC8345204; doi:10.3390/cancers13153702)
Supplement: Supplementary file 1 [file cancers-13-03702-s001.zip › cancers-1278648-supplementary.pdf]

Supplementary materials

Serum sCD25 Protein as a Predictor of Lack of Long-Term Benefits from Immunotherapy in Non-Small Cell Lung Cancer: A Pilot Study

Anna Siemiątkowska, Maciej Bryl, Katarzyna Kosicka-Noworzyń, Jakub Tvrdoň, Iwona Gołda-Gocka, Aleksander Barinow-Wojewódzki, Franciszek K. Główka

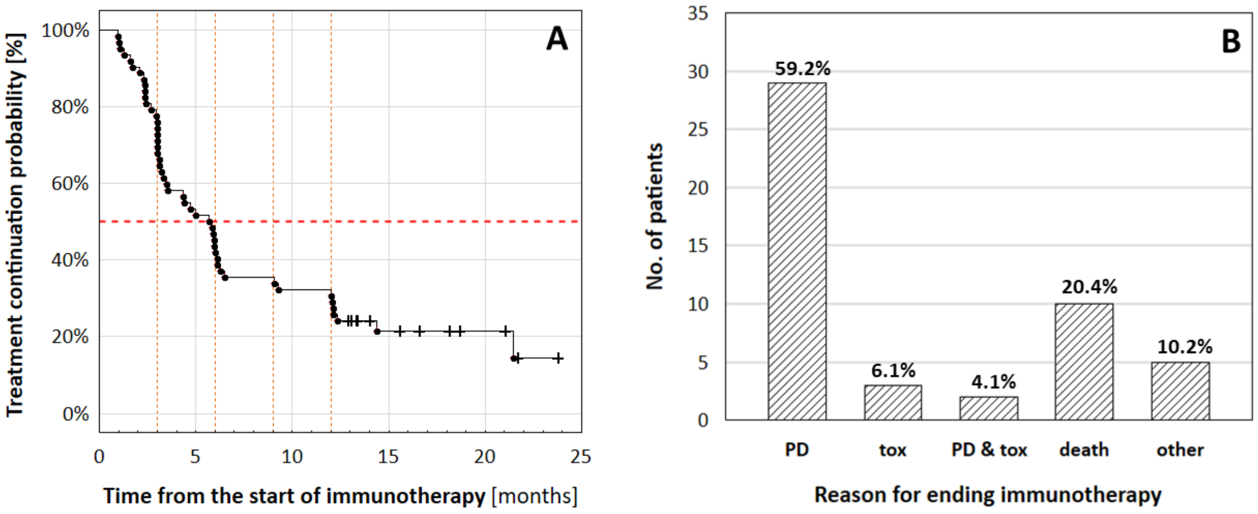

Figure S1. (**A**) Time to treatment failure and (**B**) reason for ending the anti-PD-1/PD-L1 treatment in patients with non-small cell lung carcinoma dosed with atezolizumab or pembrolizumab. Abbreviations: PD – progressive disease, tox – serious toxicity (grade ≥3).

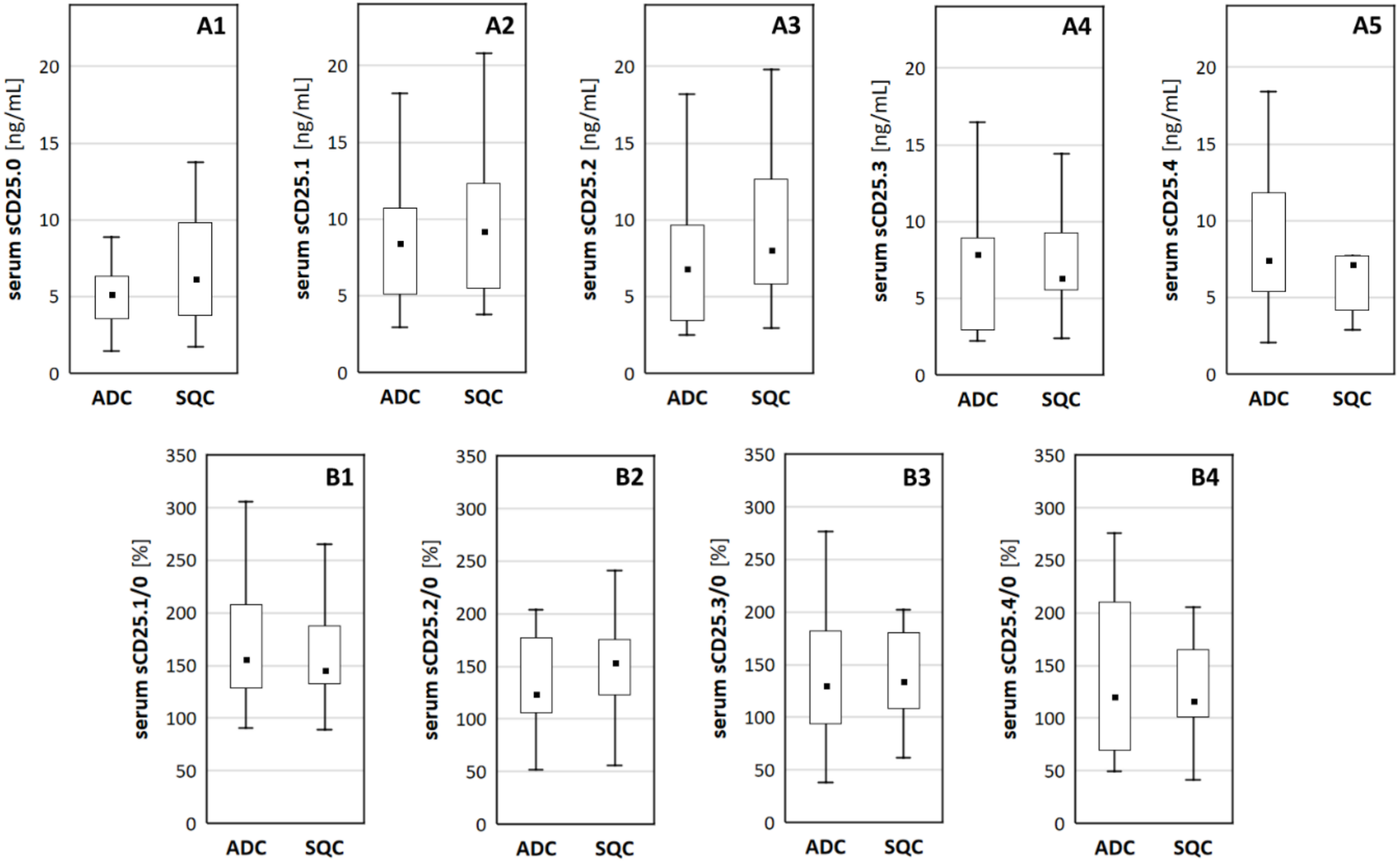

Figure S2. Serum sCD25 levels and their relative changes stratified by NSCLC subtype. Figures A1 – A5 present sCD25 (**A1**) at baseline and at the end of (**A2**) cycle 1, (**A3**) cycle 2, (**A4**) cycle 3, (**A5**) cycle 4; figures B1 – B4 present the relative changes in sCD25 compared to baseline at the end of (**B1**) cycle 1, (**B2**) cycle 2, (**B3**) cycle 3, (**B4**) cycle 4 in patients with adenocarcinoma (ADC) and squamous cell carcinoma (SQC) treated with the anti-PD-1/PD-L1s (atezolizumab or pembrolizumab). The Mann-Whitney U test revealed no significant differences in tested parameters between subjects with different subtypes of NSCLC. Box-plots show medians (filled squares), interquartile ranges (boxes), and ranges excluding outliers.

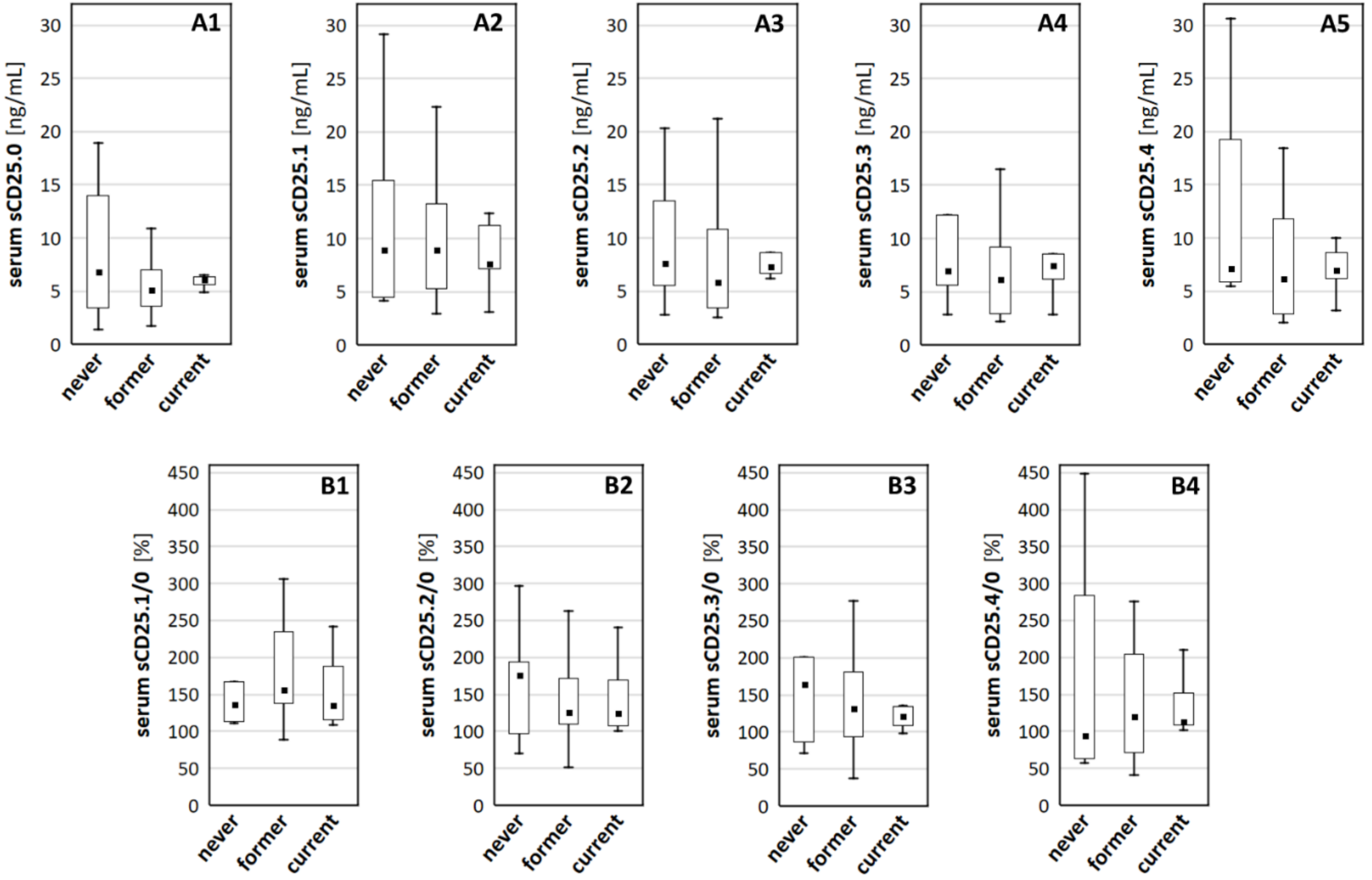

Figure S3. Serum sCD25 levels and their relative changes stratified by smoking status. Figures A1 – A5 present sCD25 (**A1**) at baseline and at the end of (**A2**) cycle 1, (**A3**) cycle 2, (**A4**) cycle 3, (**A5**) cycle 4; figures B1 – B4 present the relative changes in sCD25 compared to baseline at the end of (**B1**) cycle 1, (**B2**) cycle 2, (**B3**) cycle 3, (**B4**) cycle 4 in patients treated with the anti-PD-1/PD-L1s (atezolizumab or pembrolizumab) who smoked <100 (never smokers) or ≥100 cigarettes in their lifetime (former and current smokers). The Kruskal-Wallis test revealed no significant differences in tested parameters between groups. Box-plots show medians (filled squares), interquartile ranges (boxes), and ranges excluding outliers.

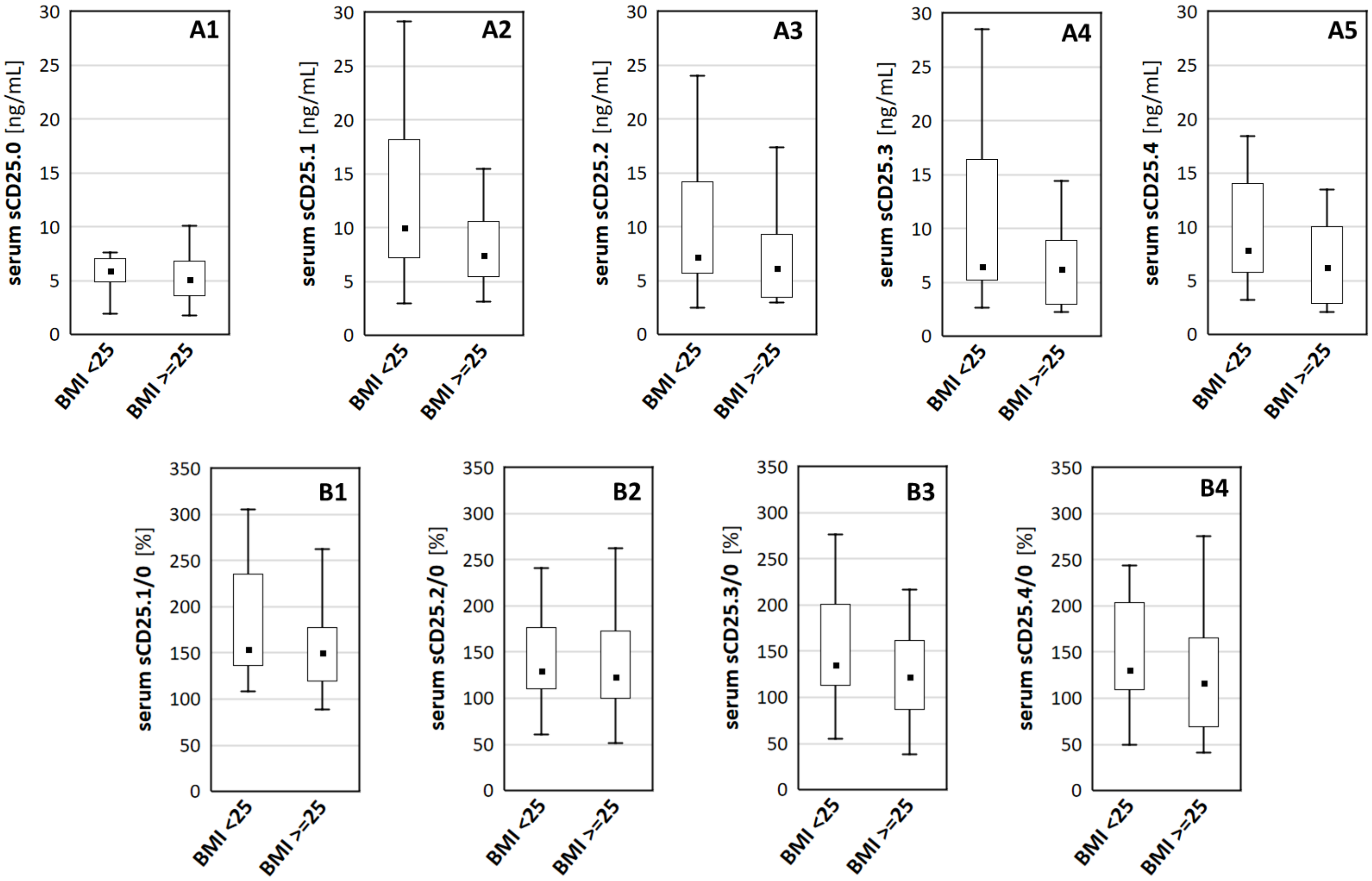

Figure S4. Serum sCD25 levels and their relative changes stratified by BMI. Figures A1 – A5 present sCD25 (**A1**) at baseline and at the end of (**A2**) cycle 1, (**A3**) cycle 2, (**A4**) cycle 3, (**A5**) cycle 4; figures B1 – B4 present the relative changes in sCD25 compared to baseline at the end of (**B1**) cycle 1, (**B2**) cycle 2, (**B3**) cycle 3, (**B4**) cycle 4 in patients with BMI < or ≥25 treated with the anti-PD-1/PD-L1s (atezolizumab or pembrolizumab). The Mann-Whitney U test revealed no significant differences in tested parameters between subjects with low and high BMI. Box-plots show medians (filled squares), interquartile ranges (boxes), and ranges excluding outliers.

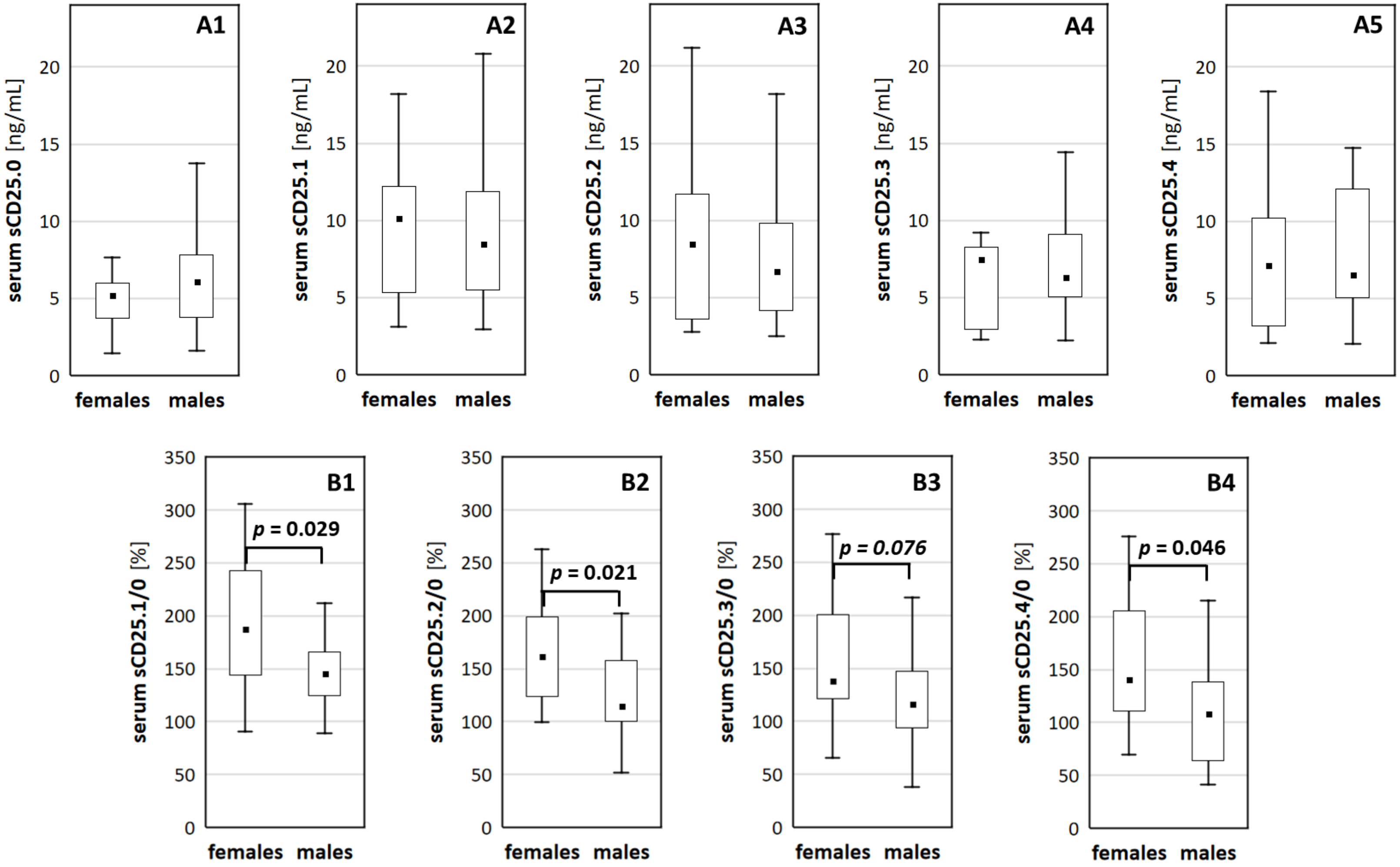

Figure S5. Serum sCD25 levels and their relative changes stratified by gender. Figures A1 – A5 present sCD25 (**A1**) at baseline and at the end of (**A2**) cycle 1, (**A3**) cycle 2, (**A4**) cycle 3, (**A5**) cycle 4; figures B1 – B4 present the relative changes in sCD25 compared to baseline at the end of (**B1**) cycle 1, (**B2**) cycle 2, (**B3**) cycle 3, (**B4**) cycle 4 in females and males treated with the anti-PD-1/PD-L1s (atezolizumab or pembrolizumab). After the start of immunotherapy, sCD25 levels increased more in female compared with male subjects. Box-plots show medians (filled squares), interquartile ranges (boxes), and ranges excluding outliers.

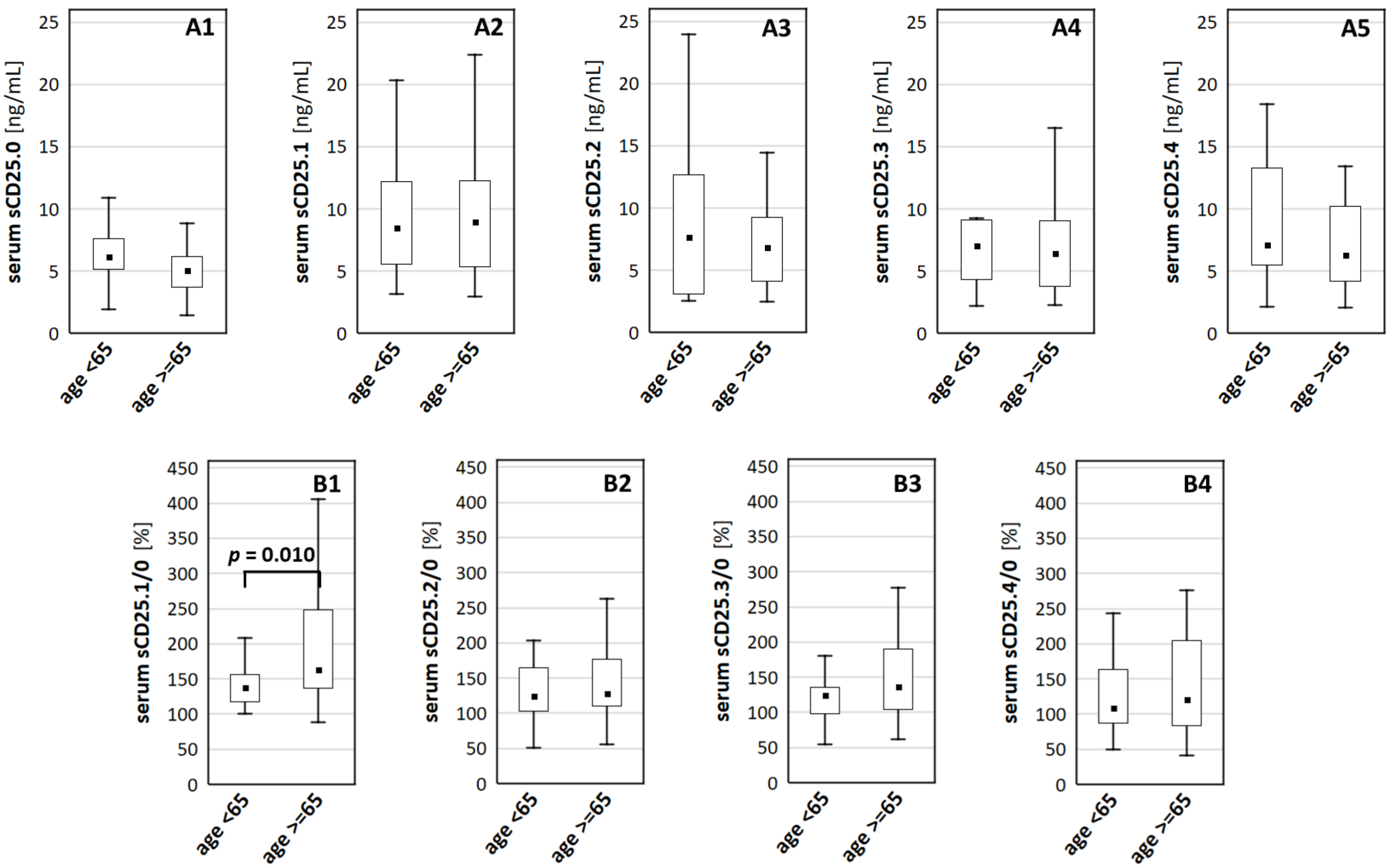

Figure S6. Serum sCD25 levels and their relative changes stratified by age. Figures A1 – A5 present sCD25 (**A1**) at baseline and at the end of (**A2**) cycle 1, (**A3**) cycle 2, (**A4**) cycle 3, (**A5**) cycle 4; figures B1 – B4 present the relative changes in sCD25 compared to baseline at the end of (**B1**) cycle 1, (**B2**) cycle 2, (**B3**) cycle 3, (**B4**) cycle 4 in younger and older patients treated with the anti-PD-1/PD-L1s (atezolizumab or pembrolizumab). At the end of cycle 1, sCD25 levels increased more in older (≥65) than in younger (<65) subjects. Box-plots show medians (filled squares), interquartile ranges (boxes), and ranges excluding outliers.

Table S1. Eligibility criteria for treatment with ATEZO or PEMBRO, according to the NSCLC drug program in Poland at the time of conducting the study (patients were recruited between February 2019 and March 2020).

I. Qualification criteria	
Criteria for qualifying patients with non-small cell lung cancer for the **first-line treatment** (patients not previously subjected to systemic treatment due to advanced cancer) with **pembrolizumab** (PDL1 expression ≥ 50%):	Criteria for qualifying patients with non-small cell lung cancer for the **second-line treatment** (patients with failure of prior multidrug platinum-based chemotherapy or monotherapy for advanced cancer) with **atezolizumab** (regardless of the PDL1 expression):
• histological or cytological diagnosis of squamous or non-squamous NSCLC;	• histological or cytological diagnosis of squamous or non-squamous NSCLC;
• presence of PDL1 expression in ≥ 50% of neoplastic cells confirmed by a method indicated in the Summary of Product Characteristics or using the DAKO 22C3 antibody concentrate or Ventana SP263 antibody;	
• no mutations in the EGFR gene and no rearrangements of the ALK gene in the case of adenocarcinoma, large-cell carcinoma, or NOS; analysis must be performed with the use of validated test and in the certified laboratory (European program for quality control);	• no mutations in the EGFR gene and no rearrangement of the ALK gene in the case of adenocarcinoma, large-cell carcinoma, or NOS; analysis must be performed with the use of validated test and in the certified laboratory (European program for quality control);
• clinical stage IV;	• locally advanced cancer (grade III - except for the cases where radiochemotherapy, radiotherapy, or surgical treatment is possible) or generalized cancer (grade IV);
• presence of measurable lesions enabling an objective assessment of the response to therapy in imaging tests, according to the RECIST 1.1 evaluation criteria, or presence of quantifiable non-measurable lesions;	• presence of measurable lesions enabling an objective assessment of the response to therapy in imaging tests, according to the RECIST 1.1 evaluation criteria, or the presence of quantifiable non-measurable lesions;
• absence of metastases in the central nervous system (CNS) or signs of progression of metastases in the CNS in patients after previous local treatment (surgery or radiotherapy), and absence of clinically significant neurological symptoms, and the need to increase the dose of glucocorticoids in the last month prior to enrollment in the program;	• no symptomatic metastases in the CNS or signs of progression of CNS metastases in patients after previous local treatment (surgery or radiotherapy), and absence of clinically significant neurological symptoms and the need to increase the dose of glucocorticoids in the last month prior to enrollment in the program;
• age over 18;	• age over 18;

• performance status of 0-1, according to the Zubrod-WHO or ECOG classification;<br>• exclusion of the coexistence of clinically significant diseases, which are impossible to control with appropriate treatment;<br>• absence of active autoimmune diseases, excluding type 1 diabetes, hypothyroidism during hormonal supplementation, psoriasis, and vitiligo;<br>• activity of the hematopoietic system enabling the treatment in accordance with the current Summary of Product Characteristics;<br>• renal function allowing the treatment (creatinine levels up to 1.5 times the upper limit of normal);<br>• liver function allowing the treatment:<br>○ bilirubin concentration not exceeding 1.5 times the upper limit of the reference range,<br>○ activity of transaminases and alkaline phosphatase not exceeding 5 times the upper limit of the reference range;<br>• absence of contraindications to the use of pembrolizumab specified in the Summary of Product Characteristics;<br>• exclusion of the simultaneous use of chemotherapy and molecularly targeted drugs;<br>• exclusion of the coexistence of other malignant neoplasms treated with a palliative approach (regardless of the response obtained) and failure to obtain a complete response in the case of neoplasms treated with a radical approach.<br><br>The eligibility criteria must be met cumulatively.	• performance status of 0-1, according to the Zubrod-WHO or ECOG classification;<br>• exclusion of the coexistence of clinically significant diseases, which are impossible to control with appropriate treatment;<br>• absence of active autoimmune diseases, excluding type 1 diabetes, hypothyroidism during hormonal supplementation, psoriasis, and vitiligo;<br>• the activity of the hematopoietic system enabling treatment in accordance with the current Summary of Product Characteristics;<br>• renal function allowing the treatment (creatinine levels up to 1.5 times the upper limit of normal);<br>• liver function allowing the treatment:<br>○ bilirubin concentration not exceeding 1.5 times the upper limit of the reference range,<br>○ activity of transaminases and alkaline phosphatase not exceeding 5 times the upper limit of the reference range;<br>• absence of contraindications to the use of atezolizumab specified in the Summary of Product Characteristics;<br>• exclusion of the simultaneous use of chemotherapy and molecularly targeted drugs;<br>• resolution or reduction to grade 1 of adverse reactions associated with previous treatment (exception: hair loss);<br>• exclusion of the coexistence of other malignant neoplasms treated with a palliative approach (regardless of the response obtained) and failure to obtain a complete response in the case of neoplasms treated with a radical approach.<br><br>The eligibility criteria must be met cumulatively.

II. Treatment duration within the program

Treatment continues until the main physician decides to exclude the patient from the program in accordance with:

- rules specified below (points II.1-II.3)
- exclusion criteria

II.1. use of pembrolizumab (1st line of treatment) or atezolizumab (2nd line of treatment) is conducted until disease progression or serious adverse effects. During therapy with the aforementioned drugs, it is possible to timely suspend the therapy due to:

a. pneumonia (grade 2 or higher);
b. diarrhea or symptoms of colitis (grade 2 or higher);
c. activity of transaminases exceeding 3 times but not more than 5 times the upper limit of the reference range, or total concentration of bilirubin exceeding 1.5 times but not more than 3 times the upper limit of the reference range;
d. concentration of creatinine exceeding 1.5 times but not more than 6 times the upper limit of the reference range, or a 1.5-fold increase of creatinine concentration with regard to the value measured at enrollment;
e. any other side effects in grade 3 or higher

II.2. during treatment, it is necessary to perform imaging tests (additional imaging tests are performed depending on the clinical situation):

a. before treatment - not earlier than 28 days before the first dose of the drug;
b. during treatment - every 3 months (test validity - 14 days)

II.3. imaging tests include assessment according to the RECIST 1.1 criteria:

a. primary lesion - computed tomography (CT) examination of the chest with epigastric extension (if the primary lung tumor does not exist due to lung parenchymal resection or the diagnosis of lung cancer was made without establishing the primary tumor, then it is necessary to demonstrate in imaging studies the metastatic measurable lesions or quantifiable unmeasurable lesions);
b. metastatic lesions - CT or other imaging tests (e.g., ultrasound, magnetic resonance - MRI, bone scintigraphy, etc.); imaging examinations during treatment should include the assessment of lesions identified before treatment initiation.

III. Exclusion criteria

III.1. disease progression confirmed by physical examination and/or imaging assessed according to the RECIST 1.1 criteria:

a. augmentation of existing lesions by at least 20%, or

b. appearance of at least one new lesion);

III.2. cancer-related deterioration of the patient's condition (clinically significant) without progression confirmed by physical examination or imaging;

III.3. occurrence of clinically significant toxicity of treatment or occurrence of at least one adverse reaction that is life-threatening according to the criteria of CTC-AE, version 4.03 (common terminology criteria for adverse events - version 4.03);

III.4. development of recurrent or unacceptable grade 3 or 4 toxicities according to CTC-AE version 4.03 (resumption of treatment is possible after resolution of symptoms of toxicity, or recovery to grade 1 or 2 according to the criteria of CTC-AE version 4.03);

III.5. hypersensitivity to the drug or the excipient;

III.6. occurrence of autoimmune organ inflammation with a severity higher than those mentioned in section II.1;

III.7. decrease in performance status to 3-4 according to WHO or ECOG criteria;

III.8. significant deterioration of quality of life as assessed by a physician;

III.9. withdrawal of consent to participate in the program (patient resignation).
